# Calcium Current Inactivation Rather than Pool Depletion Explains Reduced Exocytotic Rate with Prolonged Stimulation in Insulin-Secreting INS-1 832/13 Cells

**DOI:** 10.1371/journal.pone.0103874

**Published:** 2014-08-08

**Authors:** Morten Gram Pedersen, Vishal Ashok Salunkhe, Emma Svedin, Anna Edlund, Lena Eliasson

**Affiliations:** 1 Islet Cell Exocytosis, Lund University Diabetes Centre, Department of Clinical Sciences Malmö, Lund University, Malmö, Sweden; 2 Center for Infectious Medicine, Department of Medicine, The Karolinska Institute, Huddinge University Hospital, Stockholm, Sweden; UPR 3212 CNRS -Université de Strasbourg, France

## Abstract

Impairment in beta-cell exocytosis is associated with reduced insulin secretion and diabetes. Here we aimed to investigate the dynamics of Ca^2+^-dependent insulin exocytosis with respect to pool depletion and Ca^2+^-current inactivation. We studied exocytosis, measured as increase in membrane capacitance (ΔC_m_), as a function of calcium entry (*Q*) in insulin secreting INS-1 832/13 cells using patch clamp and mixed-effects statistical analysis. The observed linear relationship between ΔC_m_ and *Q* suggests that Ca^2+^-channel inactivation rather than granule pool restrictions is responsible for the decline in exocytosis observed at longer depolarizations. INS-1 832/13 cells possess an immediately releasable pool (IRP) of ∼10 granules and most exocytosis of granules occurs from a large pool. The latter is attenuated by the calcium-buffer EGTA, while IRP is unaffected. These findings suggest that most insulin release occurs away from Ca^2+^-channels, and that pool depletion plays a minor role in the decline of exocytosis upon prolonged stimulation.

## Introduction

Insulin is secreted from the pancreatic beta-cells following an increase in glucose concentration to mediate uptake of glucose into target tissue. Failure of the beta-cells to release enough insulin is an important factor in the development of type-2 diabetes. Recent genetic data have demonstrated that the expression of genes involved in the final steps of insulin secretion is reduced in patients with type-2 diabetes [1], [2]. These final steps include influx of Ca^2+^ through voltage-dependent Ca^2+^ channels triggering exocytosis of insulin containing granules and release of insulin [3]. Moreover, four of the top genetic risk variants associated with human type-2 diabetes is associated with reduced exocytosis [4]. A better understanding of the exocytotic process will consequently have important clinical implications.

As mentioned, the release of insulin from the secretory granules is a result of calcium-triggered exocytosis, which follows Ca^2+^ influx through voltage-gated channels [5], [6]. Such exocytosis can be measured as an increase in the cell membrane capacitance (ΔC_m_) using the voltage-clamp mode of the patch-clamp technique [7]. Since the membrane capacitance cannot be measured reliably during a depolarization, voltage pulses of different durations, the so-called pulse-length protocol, have been applied to study the kinetics of insulin exocytosis [6], [8]–[12].

In murine beta-cells, the rate of exocytosis is typically higher in response to shorter than to longer depolarizations, resulting in a biphasic capacitance pattern that has been suggested to correspond to biphasic insulin secretion, and to be caused by depletion of an immediately releasable pool (IRP) of granules located near Ca^2+^ channels [8], [10], [11], [13], [14]. A similar decline of the exocytotic response is seen in rat beta-cells [15] and rat insulinoma insulin-secreting INS-1 cells [16]. The IRP is a sub-pool of the larger readily releasable pool (RRP), which contains all granules that can be released by flash-release of caged-Ca^2+^ [8], [17]. However, due to inactivation of Ca^2+^ currents, Ca^2+^ influx shows a biphasic pattern resembling the biphasic exocytotic response [6], [15], and hence current inactivation, rather than IRP depletion, has also been suggested to cause the decline in the exocytotic pattern in response to depolarizations of increasing lengths [6], [18].

In isolated human beta-cells the rate of the exocytotic response does not decrease but rather increases with prolonged stimulation [9], [19]. We have suggested in a theoretical study that this is because of the absence of an IRP, and that granules are located away from Ca^2+^ channels in single human beta-cells [20]. The situation is different in human islets, where beta-cells *in situ* show a decline in the exocytotic response [21].

Recently, a detailed theoretical study showed that to investigate whether pool depletion occurs, depolarization-evoked exocytosis should be studied as a function of Ca^2+^ influx rather than of time [22]. In general, only a clear deviation from a linear relation between the depolarization-evoked Ca^2+^ influx, *Q*, and the resulting increase in capacitance, ΔC_m_, suggests pool depletion [22]. Interesting, several studies report a linear relation between *Q* and ΔC_m_ in beta-cells [8], [10], [15], a finding that we recently confirmed by mixed-effects statistical analysis of pulse-length data obtained from mouse beta-cells by the perforated-patch technique [20]. In contrast, the exocytotic response of human beta-cells in intact islets plateaus when analyzed as a function of *Q* [21], hence exhibiting the characteristic pattern of pool depletion [22].

Mixed-effects statistical modeling [23] is appropriate for studying clustered data, e.g., when several depolarizations are applied to the same cell, as in the case of the pulse-length protocol. We would expect results from a single cell to be more closely correlated than data from different cells. Pooling of data from different cells treats cell-to-cell variation and experimental errors equally, and neglects natural cell heterogeneity. Mixed-effects modeling can handle and quantify such biological variation while at the same time account for within-cell correlation.

Here we investigated the exocytotic response in INS-1 832/13 to obtain a deeper understanding of the dynamics of exocytosis. The INS-1 832/13 cells are rat insulinoma cells expressing human insulin with good glucose responsiveness [24], which are often utilized to investigate the physiological role of different human genetic findings [25]–[28]. We have applied different depolarization protocols and mixed-effects modeling of ΔC_m_ as a function of *Q* to investigate whether depletion of a pool of granules underlies the decline of the capacitance pattern in response to depolarizations of different lengths in INS-1 832/13 cells. We find no evidence of pool depletion contributing to the exocytotic profile and suggest that the reduced rate of exocytosis is due to inactivation of the Ca^2+^ current. Inclusion of high concentrations of the Ca^2+^ buffer ethylene glycol tetraacetic acid (EGTA) does not interfere with the IRP but lowers the sensitivity of later exocytosis to Ca^2+^ entry, indicating that later fusion occurs away from Ca^2+^ channels.

## Methods

### Cells and cell culture

Rat insulinoma INS 832/13 cells [24] were grown in 10-cm tissue culture dishes at 37 °C and 5% CO_2_ and cultured in RPMI 1640 media (ThermoScientific, Hyclone Laboratories Inc, Utah, US) with 11.1 mM glucose and supplemented with 10% FBS (wt/vol), 100 U/ml penicillin, 0.1 mg/ml streptomycin, 10 mM HEPES, 2 mM L-glutamine, 1 mM sodium pyruvate, and 50 µM β-mercaptoethanol. At ∼80% confluence the cells were split 1∶8 using Trypsin-EDTA and seeded into 35 mm petri dishes, where they were left overnight prior to patch-clamp experiments.

### Electrophysiology

Whole cell currents and exocytosis were recorded using an EPC-9 patch-clamp amplifier (HEKA electronics; http://www.heka.com) and the software Pulse (ver 8.80, HEKA electronics). Exocytosis was recorded as changes in membrane capacitance using the standard whole-cell configuration of the patch-clamp technique.

The extracellular medium contained the following (in mM): 118 NaCl, 20 tetra-ethyl-ammonium chloride (TEA-Cl; to block voltage-gated K^+^-currents), 5.6 KCl, 2.6 CaCl_2_,1.2 MgCl_2_, 5 glucose, and 5 HEPES (pH 7.4 using NaOH). The standard pipette solution (IC1) consisted of the following (in mM): 125 Cs-glutamate, 10 NaCl, 10 CsCl, 1 MgCl_2_, 0.05 EGTA, 3 Mg-ATP, 0.1 cAMP and 10 HEPES (pH 7.15 with CsOH). In other experiments Ca^2+^ buffering was increased by including 10 mM EGTA and 2.5 (IC2) or 7 mM CaCl_2_ (IC3), respectively. The free Ca^2+^ concentration was estimated using the software Maxchelator (Ca-Mg-ATP-EGTA Calculator v1.0; http://maxchelator.stanford.edu; [29]) to be ∼60 and ∼460 nM, and the calculated free EGTA concentration was ∼7.0 and ∼2.8 mM for IC2 and IC3, respectively. Patch electrodes were pulled from borosilicate capillaries, coated with Sylgard (Dow Corning Midland), and fire polished. The pipette resistance was 3–7 MΩ when the pipettes were filled with intracellular solution.

Exocytosis was detected as changes in membrane capacitance using the software-based lock-in application (which adds a sine wave of 500–1,000 Hz to the holding potential) of the amplifier. Exocytosis was elicited by depolarizations from −70 to 0 mV at varying pulse durations (5, 10, 20, 40, 80, 160, 320 and 640 ms; standard pulse length protocol). In another series exocytosis was evoked by a double pulse protocol, meaning that two 50-ms depolarizations from −70 to 0 mV separated by a 100-ms interval were applied. The double pulse experiments were part of a larger protocol where variations of the double-pulse and pulse-length protocols were used. In this larger series all depolarizations of varying pulse duration (50, 100, 200, 400 and 800 ms) were preceded by a 50-ms depolarization from −70 mV to 0 mV. The interval between the depolarization of varying length and the pre-pulse was 100 ms. In addition, a protocol using the combined pre-pulse pulse length protocol was used and each pair of depolarizations was followed by a third 500-ms depolarization from −70 to 0 mV applied either 200 ms (protocol I) or 10 s (protocol II) after the end of the second pulse. Finally, the responses to a train of ten 500 ms depolarizations delivered at 1 Hz were studied (train protocol). Pulses of different lengths were given in varying order, and no dependency on the order of the pulses was found. All experiments were conducted at 30–32°C.

### Data analysis

Ca^2+^-currents were inspected visually, and data with large leak currents or high amounts of noise were discarded for the analysis. An in-house MATLAB (Mathworks Inc.) script was used to extract total calcium influx, *Q*, and increase in capacitance, ΔC_m_, for each experiment (available within Data S1–S4). Evoked exocytosis ΔC_m_ was set in relation to *Q* to avoid the complication of Ca^2+^ current inactivation [22].

In a first statistical analysis, we fitted linear models for regression of ΔC_m_ on *Q*, taking the differences between cells into account. As in our previous studies [20], [30] a significant between-cell variation was present, and we proceeded by fitting linear mixed-effects models [23], which are more appropriate for representing clustered data, such as in our case where several observations are done on each cell.

The linear mixed-effects models included treatment group as fixed effect and cell as random effect. The appropriate mixed-effects model for each experimental protocol was found by a step-wise procedure; where at each step the non-significant term with the highest p-value was excluded. We verified at each step that the simplified model was preferable to the larger model by a likelihood ratio test and the Akaike Information Criterion.

We found no statistical differences between the groups with high EGTA concentrations and ∼60 nM or ∼460 nM free Ca^2+^ for any of the protocols. Hence, they were considered as a single treatment group (“EGTA”) to be compared to the control (“CTRL“) group for the final analyses.

The final model for analysis of the standard pulse-length protocol with depolarizations of 5, 10, 20, 40, 80, 160, 320 and 640 ms was of the form
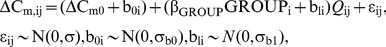
(1)


for observation j of cell i, where ΔC_m0_ is the intercept representing an increase in capacitance in the limit of zero Ca^2+^ entry, β_GROUP_ ( =  β_CTRL_, β_EGTA_) is the slope of the relation between *Q* and ΔC_m_ representing the efficacy of Ca^2+^ entry in the control and EGTA groups. Moreover, ε_ij_ is a normally distributed error term, GROUP_i_ is a covariate indicating whether cell i was in the “CTRL“ or “EGTA” group, b_0i_ and b_1i_ are factors allowing for cell-to-cell variation, and the parameters ΔC_m0_, β_CTRL_, β_EGTA_, σ, σ_b0_ and σ_b1_ are to be estimated.

The mixed-effects model describing the data from the two 50 ms pulses of the double-pulse protocol was 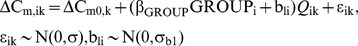
(2)


for pulse k = 1,2 of cell i. Parameters are as in Eq. 1, except that the intercept (ΔC_m0,k_) is allowed to differ between the two pulses.

For the experiments where each pulse of a pulse-length protocol (50, 100, 200, 400 and 800 ms depolarizations) was preceded by a 50 ms prepulse, the data from the pulses of varying lengths following the prepulse were well-described by a model with no intercept of the form
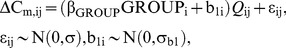
(3)


for observation j of cell i, Parameters are as in Eq. 1.

The final model for the data from the third pulses, following 50 ms prepulses and pulses of varying length (200, 400 or 800 ms), and either 200 ms (protocol I) or 10 s (protocol II) rest, did not include any effect of the protocol, 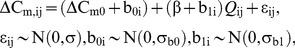
(4)


for observation j of cell i, Parameters are as in Eq. 1, except that the Ca^2+^ current sensitivity β was the same for all cells, since these protocols were only applied under control conditions.

Data from the train protocol was analyzed by relating cumulative increase in membrane capacitance, (ΣΔC_m_)_ij_  =  ΔC_m,i1_ +… + ΔC_m,ii_, to cumulative calcium entry, (Σ*Q*)_ij_  =  *Q*_i1_ +… + *Q*_ij_, for pulse j of cell i. The final model was similar to Eq. 4,
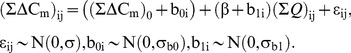
(5)


To investigate whether pool depletion occurred, we added a quadratic term γ*Q*_ij_^2^ to the models describing pulse-length data without (Eq. 1) or with (Eq. 3) a prepulse, to test whether there was a significant concave (downward curvature) deviation from linearity in the data, which would be indicative of pool depletion [22]. In no cases was the quadratic term significantly smaller than zero.

The statistical software R [31] was used for data analysis, in particular the lme function of the nlme R-package [23], [32]. Parameter estimates are given with standard errors and p-values from two-sided t-tests. P-values <0.05 were considered statistically significant.

## Results

### The linear relationship between exocytosis and Ca^2+^ influx does not deviate in presence of EGTA

**Figure 1:**
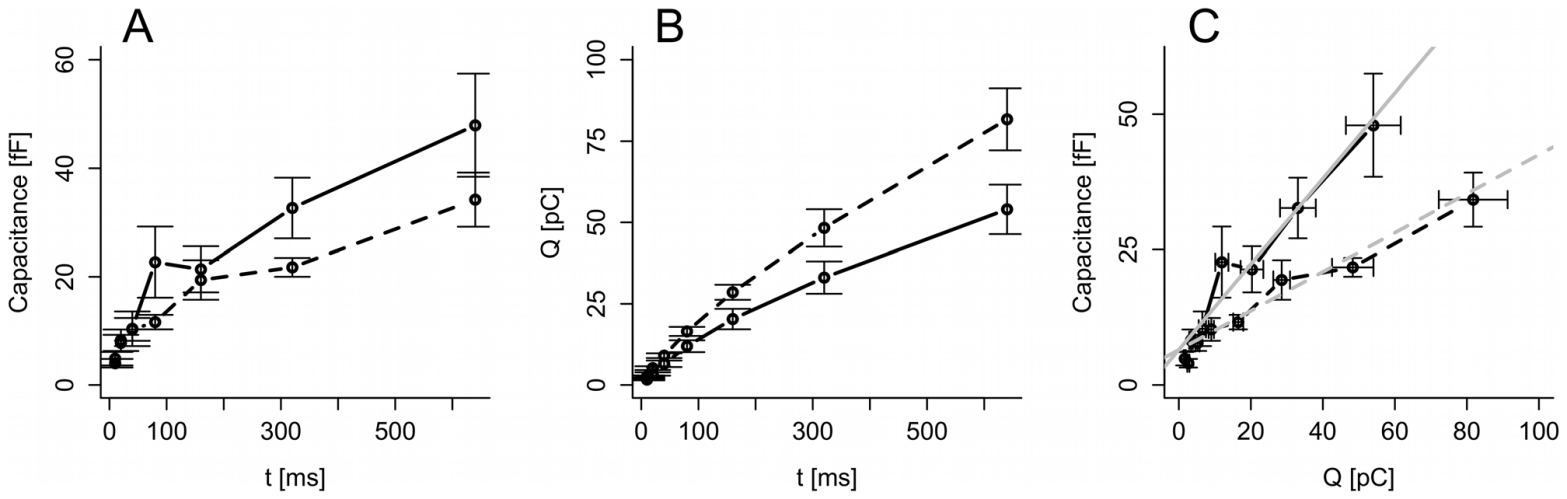
Summary of the standard pulse-length protocol data from the control (n = 17 cells; solid) and EGTA (n = 15 cells; dashed) groups. Data are means ± SEM for data pooled according to pulse-length. A: Evoked capacitance increases ΔC_m_ for each depolarization of varying length (t). B: Evoked Ca^2+^ influx *Q* for each pulse length (t). C: ΔC_m_ vs. *Q* for each pulse length. The gray lines indicate the Ca^2+^ current sensitivity in the control (solid) and EGTA (dashed) groups, as estimated by the linear mixed-effects model (see Fig. 2).

First, we were interested in investigating kinetics of exocytosis using an experimental setting used previously [8], [33]. Accordingly, single INS-1 832/13 cells were subject to capacitances measurements using the whole-cell configuration of the patch-clamp technique. Capacitance increases were evoked by the pulse-length protocol, which depolarizes the membrane potential from −70 mV to 0 mV during voltage-clamp periods of varying length. Pulses of different duration were applied in varying order, and no dependence on the order was found. The capacitance increase (ΔC_m_) reflecting exocytosis showed a biphasic relation to the pulse length such that the average rate of exocytosis was higher during short than during longer pulses (Fig. 1A) [12], [34]. This biphasic pattern has been suggested to be caused by depletion of IRP located near Ca^2+^ channels [8], [33]. However, because of Ca^2+^ current inactivation, the amount of Ca^2+^ (*Q*) that enters the cell during each depolarization does not have a simple relationship to pulse length (Fig. 1B). It might be that the biphasic pattern of the increase in membrane capacitance is caused by current inactivation rather than IRP depletion, and to investigate this question one should relate ΔC_m_ to *Q* rather than to pulse length [18], [22]. Indeed, experiments performed on INS-1 cells has previously demonstrated that a depolarization of the same size and duration (300 ms) can give rise to large differences in Ca^2+^ influx measured as charge (*Q*). Plotting ΔC_m_ to *Q* in this case gave a linear relationship [35]. Our data showed a near-linear ΔC_m_ to *Q* relation (Fig. 1C), as previously observed in mouse beta- [8], [10], [20] and alpha-cells [30], [36]. In contrast, human beta-cells *in situ* show a nonlinear, concave ΔC_m_ to *Q* relation [21].

To obtain spatial information of the exocytotic machinery, the Ca^2+^ buffer EGTA (10 mM) was included in the patch-pipette. Under these conditions the general patterns of ΔC_m_ and *Q* were unchanged, but the amount of exocytosis was reduced while Ca^2+^ entry increased. EGTA is a relatively slow Ca^2+^ buffer, and 10 mM EGTA chelates Ca^2+^ ions at a typical distance of ∼100 nm away from Ca^2+^ channels [37]. EGTA inclusion led to a reduction in the slope of ΔC_m_ as a function of *Q*, from ∼0.8 fF/pC in control cells to ∼0.3 fF/pC in the presence of intracellular EGTA. Interesting, there was no difference between the responses in groups with 10 mM EGTA and ∼60 nM or ∼460 nM free Ca^2+^, excluding that the effect of EGTA was because of lower Ca^2+^-stimulated granule recruitment due to a reduction of basal [Ca^2+^]. We therefore united the two EGTA pools in Fig. 1.

### Mixed-effect model analysis reveals unaffected IRP and reduced Ca^2+^ current sensitivity in presence of EGTA

The data representation in Fig. 1 pools the responses from different cells together, in which case natural cell-to-cell heterogeneity is neglected and considered merely as experimental errors. Responses to the various depolarizations applied to the same cell are likely correlated, while responses in different cells are not, but reflect genuine biological heterogeneity. In order to handle this scenario appropriately, we applied statistical mixed-effects modeling, as done previously in our studies of exocytosis in mouse beta- and alpha-cells [20], [30].

The data was well described by a linear mixed-effects model with fixed effects describing the Ca^2+^ current sensitivity in the control and EGTA group, respectively. We use the terminology “Ca^2+^ current sensitivity” to mean “the sensitivity of exocytosis to Ca^2+^ entry via Ca^2+^ channels”, which is different from the biochemical Ca^2+^ sensitivity of exocytosis [38].

**Figure 2:**
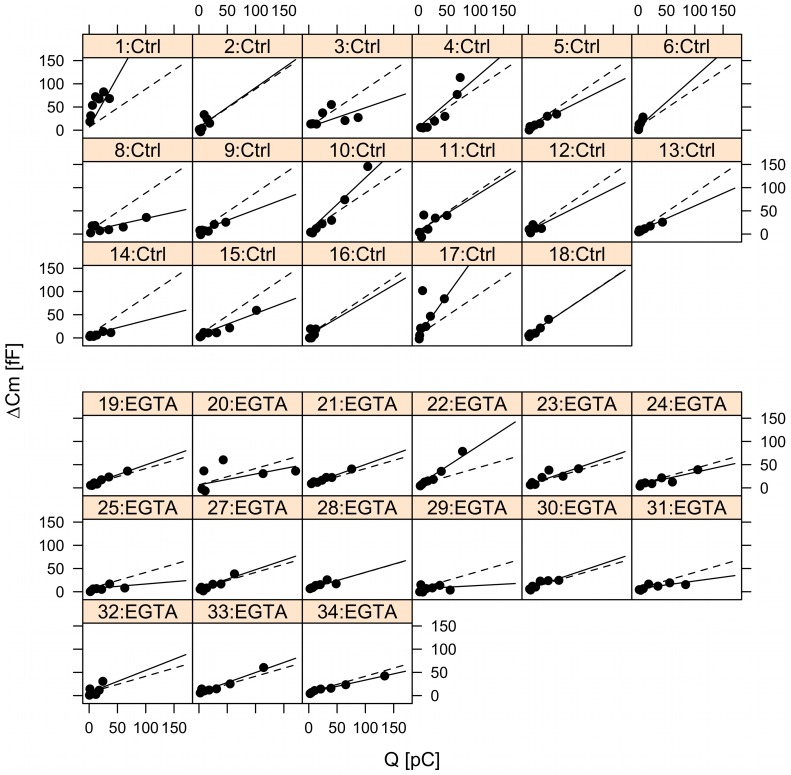
Mixed-effects analysis of pulse-length data in the control and EGTA groups. The panels show capacitance data plotted against Ca^2+^ influx measured as charge (Q) from individual cells with single-cell fits indicated by solid lines, while group fits (fixed-effects) are given by the dashed lines. Ctrl (panel 1–18), EGTA (panel 19–34).

Likelihood ratio test confirmed that the data did not show evidence of differences between the two EGTA groups with ∼60 nM or ∼460 nM free [Ca^2+^] (p = 0.89). Moreover, the data could be fitted with a model with common intercept (ΔC_m0_) for the control and EGTA groups. This intercept reflects exocytosis in the limit of zero Ca^2+^ entry. The random effects describe cell-deviation from the group estimates for the intercept and Ca^2+^ current sensitivity (Fig. 2).

The common intercept for the control and EGTA groups was estimated to be 6.5±1.6 fF, significantly larger than zero (p<0.001, n = 32 cells), corresponding to ∼10 granules based on a mean single-granule capacitance of 0.6 fF in INS-1 832/13 cells [34]. The Ca^2+^ current sensitivity was 0.79±0.10 fF/pC in the control group and it was significantly lower in the EGTA group (0.36±0.09 fF/pC; p = 0.002). Thus, EGTA interfered with granule fusion caused by larger amounts of Ca^2+^ entry, suggesting that later exocytosis occurred away from Ca^2+^ channels [37]. In contrast, increased Ca^2+^ buffering by EGTA did not interfere with the small pool, which is likely located very near Ca^2+^ channels, and corresponds to IRP. There was substantial cell-to-cell variation with estimated standard deviations of 6.7 fF and 0.32 fF/pC for the random effects of the intercept and the Ca^2+^ current sensitivity, respectively. There was no evidence for a deviation from linearity in the data (p = 0.39).

### A 50-ms pre-pulse depletes IRP but not later exocytosis

**Figure 3:**
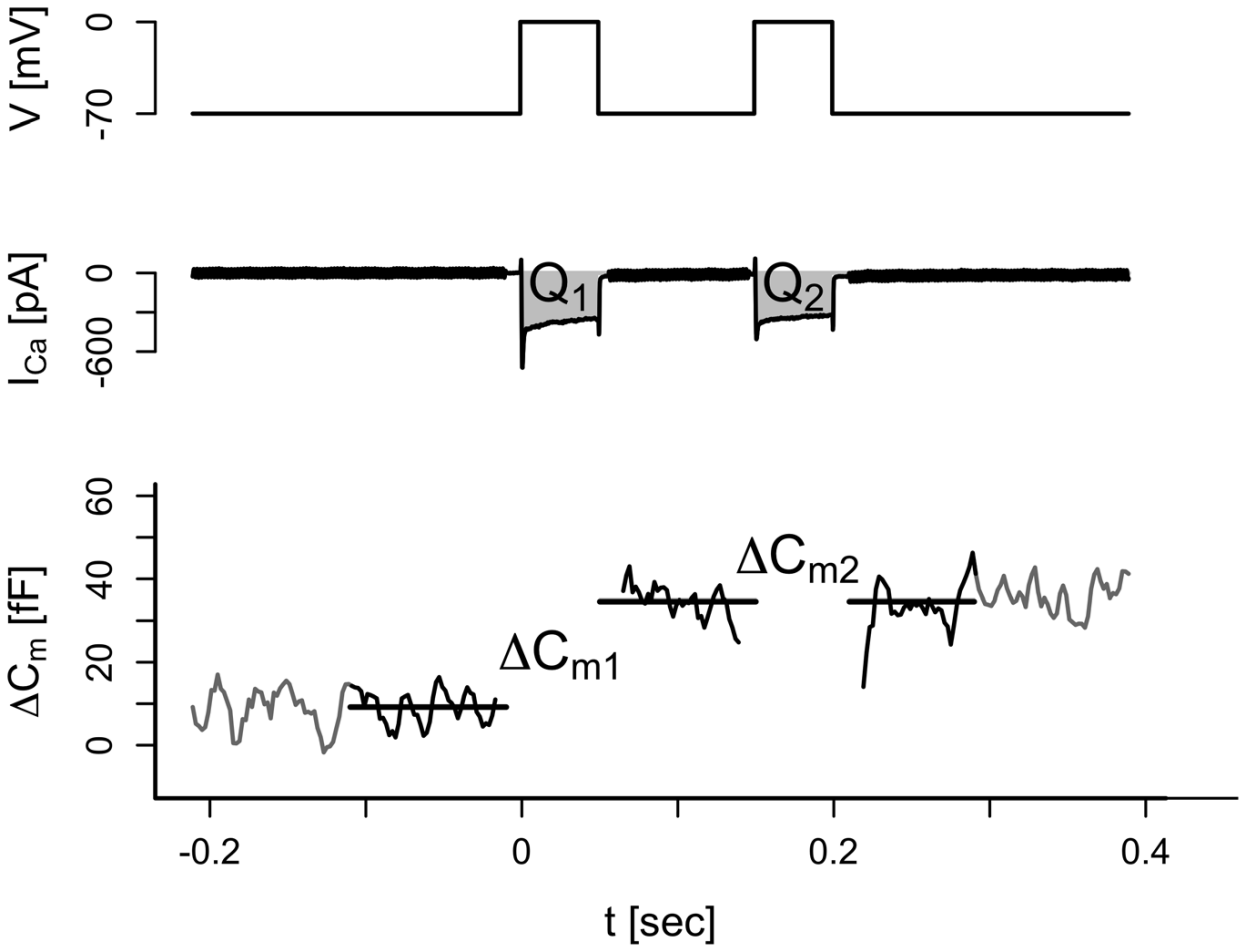
Demonstration of the double-pulse protocol. Single cells were depolarized from a holding potential of −70 mV to 0 mV during 50 ms. This was followed by a resting period of 100 ms and a second 50-ms depolarization from −70 mv to 0 mV (top trace). The evoked Ca^2+^ currents were measured and the charges for the first (Q_1_) and second (Q_2_) pulses were estimated (middle trace). In addition, the increases in membrane capacitance evoked by the first (ΔC_m1_) and second (ΔC_m2_) depolarizations were measured.

An alternative to the pulse-length protocol for the study of pool depletion is the double-pulse protocol [15], [39], where two depolarizations are applied separated by a short resting period, and the capacitance responses are measured. To investigate pool depletion from another angle we used this protocol and accordingly applied two 50 ms depolarizations to 0 mV from −70 mV separated by a 100 ms interval, in absence (control) and presence of 10 mM EGTA in the patch pipette (Fig. 3). Gillis et al. [39] suggested adjusting the voltage between pulses, such that calcium channel inactivation is balanced by stronger channel activation during the second pulse. However, the degree of inactivation is unknown until the experiment is performed, which complicates the choice of the voltage to apply during the second pulse.

**Figure 4:**
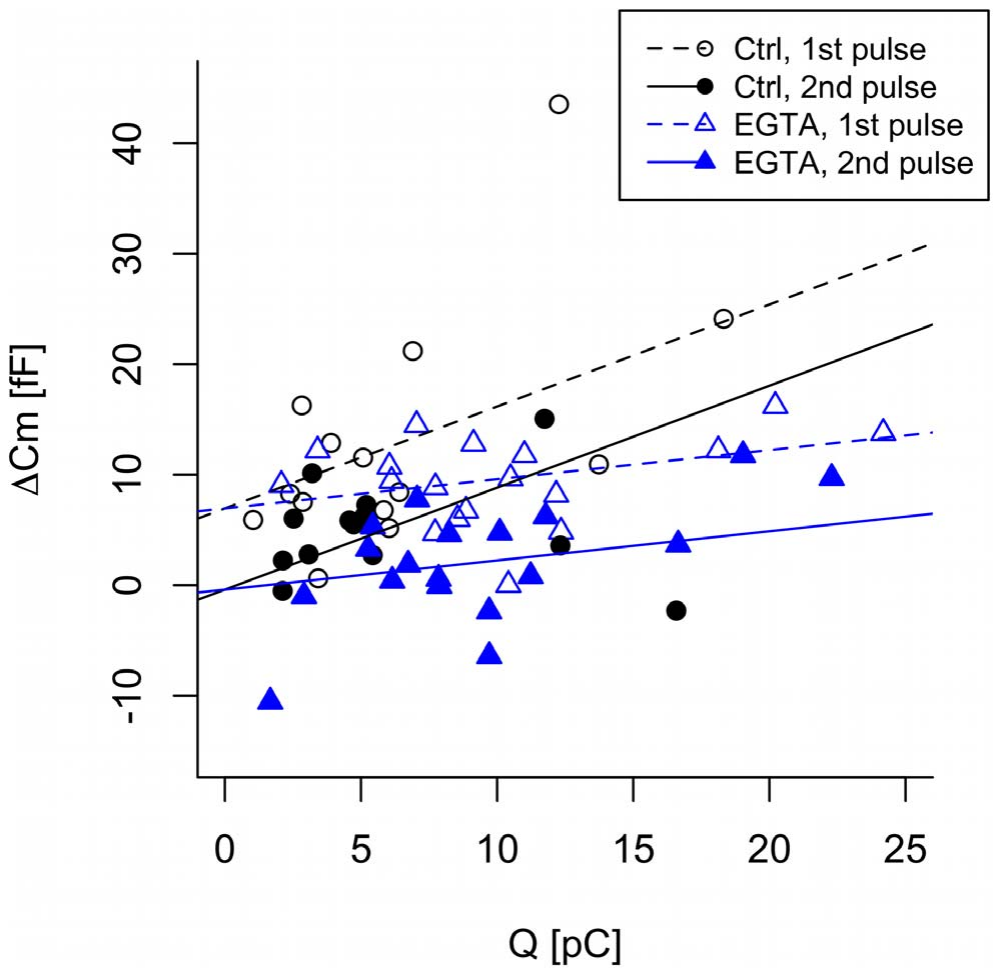
Analysis of the double-pulse data. The exocytotic response (ΔC_m_) is plotted against Ca^2+^-influx (charge; Q). Capacitance increases and Ca^2+^ influxes evoked by the first 50 ms-depolarization (Pulse 1) are shown as open symbols with fixed-effects fits indicated by dashed lines, while data evoked by the second 50-ms depolarization (Pulse 2) are plotted as filled symbols with their fixed-effects fits given by the solid lines. The colors and symbols indicate the groups (Black circles and lines: CTRL; Blue triangles and lines: EGTA). The graph contains data from n = 14 and n = 18 experiments from the control and EGTA group, respectively.

To circumvent this issue without neglecting the problem of Ca^2+^ current inactivation, we analyzed the capacitance increases ΔC_m_ as a function of Ca^2+^ entry *Q,* derived under control conditions and in the presence of EGTA. A simple linear regression model with slope depending on both the group (CTRL/EGTA) and the pulse number revealed no statistically significant dependence on the pulse number (estimated slopes for the CTRL group: 1.18±0.28 vs. 0.42±0.31 fF/pC (p = 0.075); EGTA: 0.33±0.19 vs. 0.25±0.21 fF/pC (p = 0.79)). The data from the two groups were therefore further analyzed by a linear mixed-effects model with a slope depending on whether EGTA was present or not, but independent of the pulse number. In contrast, the intercept depended on the pulse number, but was not influenced by EGTA, and therefore common for the control and EGTA groups (Fig. 4). For the first pulse, the common intercept for the two groups was larger than zero (p<0.001) and estimated to be 6.94±1.68 fF. In contrast, for the second pulse the common intercept was estimated to be −0.40±1.59 fF, not statistically different from zero (p = 0.8), showing that the first pulse depleted the small pool. EGTA lowers the Ca^2+^ current sensitivity from 0.92±0.28 fF/pC in the control group to 0.26±0.19 fF/pC in the EGTA group (p<0.001). Together, these results confirm the presence of an IRP of ∼7 fF, which is unaffected by EGTA and depleted by the first depolarization. The exocytotic response to higher amounts of Ca^2+^ entry is reduced by EGTA and similar for the first and the second pulse, indicating that the first pulse does not deplete the pool responsible for later exocytosis.

**Figure 5:**
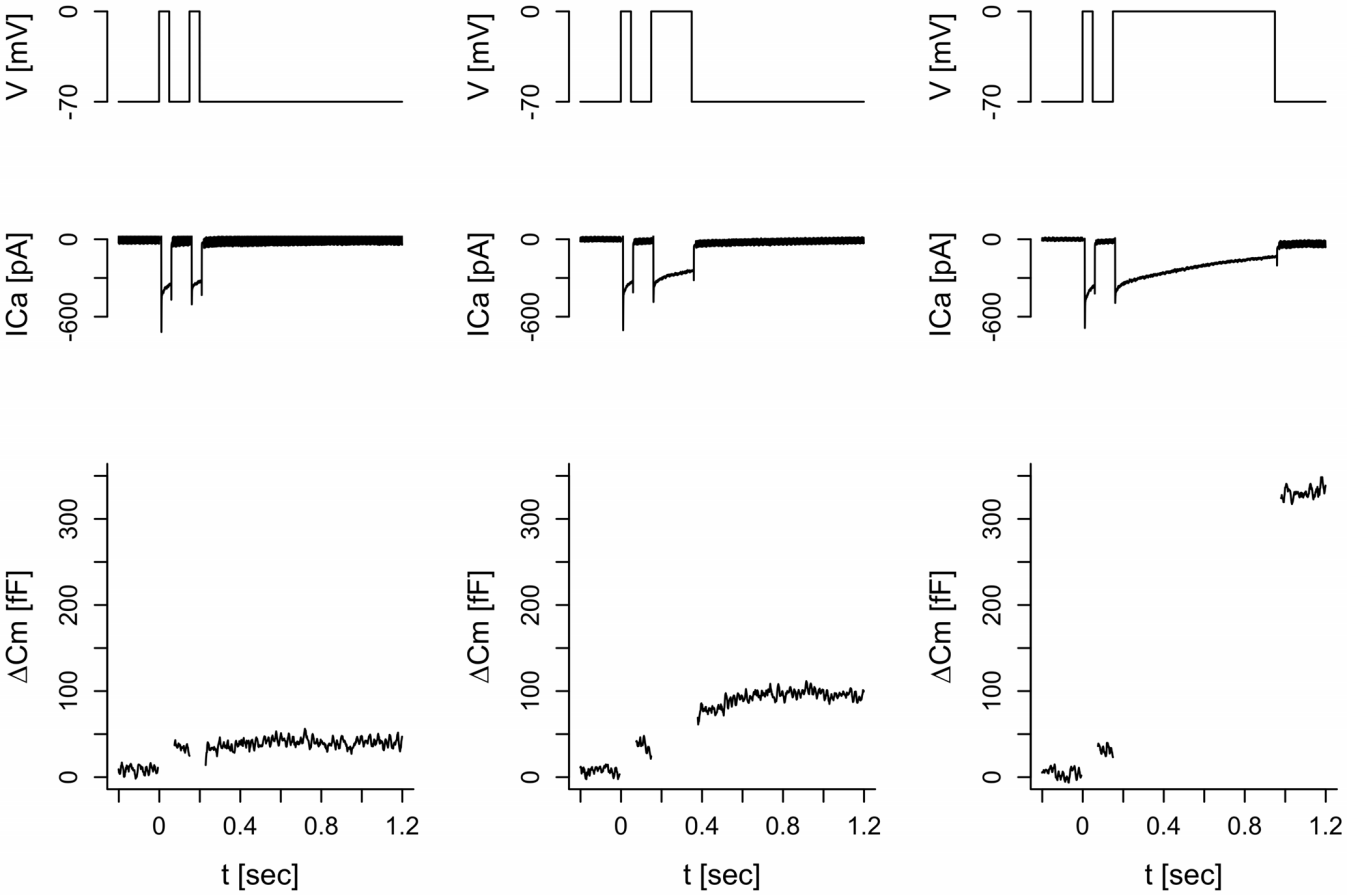
Illustration of the protocol where a 50 ms prepulse is followed by depolarizations of varying length (50, 100, 200, 400, and 800 ms). The applied changes in membrane potential due to the depolarizations (V; top) evoke Ca^2+^-currents (ICa; middle) and increases in membrane capacitance (ΔCm; bottom). The panels show the data from a single cell in response to 3 (50 ms prepulse + 50, 200 or 800 ms depolarization) of the 5 double-pulses applied to the cell. We analyzed the data from the depolarizations of varying length following the prepulse.

The double-pulse data set analyzed above is a subset of a larger dataset where 50 ms prepulses followed by 100 ms resting periods at −70 mV and depolarizations of varying lengths (50, 100, 200, 400 and 800 ms), were applied to each cell (Fig. 5).

We reasoned that if the cells possessed a limited pool corresponding to a capacitance increase of 20–30 fF (cf. Fig. 1A), then a prepulse of 50 ms evoking a capacitance increase of ∼10 fF (cf. Fig. 1A) would change the Ca^2+^ current sensitivity of the second pulses of varying length compared to the case of the pulse-length protocol with no prepulse (Figs. 1 and 2), since the inflowing Ca^2+^ would have fewer granules to act upon because of pool depletion.

**Figure 6:**
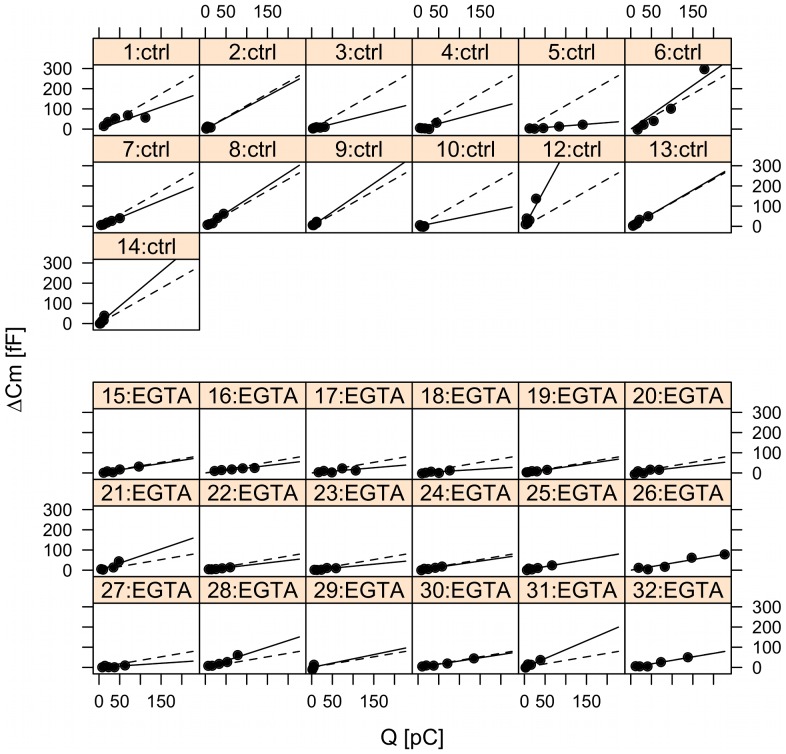
Mixed-effects analysis of the pulse-length data from the second pulse, following a 50 ms prepulse, in the control and EGTA groups. Capacitance data (ΔCm) plotted against Ca^2+^ influx measured as charge (Q) from individual cells with single-cell fits indicated by solid lines, while group fits (fixed-effects) are given by the dashed lines. Ctrl (panel 1–14); EGTA (panel 15–32).

Linear mixed-effects modeling of ΔC_m_ as a function of *Q* for the second pulse (Fig. 6) showed that the data could be fit with an intercept of zero, confirming that the prepulse depleted the small pool of ∼10 granules. The Ca^2+^ current sensitivity in the control group was 1.17±0.19 fF/pC (n = 13 cells), while it was significantly lower in the EGTA group (0.35±0.15 fF/pC, n = 18 cells; p<0.001 by t-test). Cell-to-cell variation was rather large as quantified by the standard deviation of 0.62 fF/pC for the random effect on Ca^2+^ current sensitivity. In summary, in the control group the Ca^2+^ current sensitivity was not reduced following a 50 ms prepulse (if anything, it tended to be slightly increased, 1.17±0.19 vs. 0.79±0.10 fF/pC, p = 0.088 by two-tailed t-test). Similarly, the application of a prepulse did not affect the Ca^2+^ current sensitivity in the EGTA group (0.35±0.15 vs. 0.36±0.09 fF/pC, p = 0.95). These results speak against pool depletion causing the capacitance pattern seen in Fig. 1A.

Interestingly, the ΔC_m_-*Q* relation did not show any concave deviation from linearity as expected in the absence of pool depletion, but rather a convex relation as indicated by a positive estimate (0.0013±0.0004 fF/(pC∧2), p = 0.0021, n = 31 cells) of the coefficient γ of the quadratic term (see Methods). Such a convex relation between ΔC_m_ and *Q* can arise from exocytosis occurring away from Ca^2+^ channels in a more general submembrane domain [21]. In support of this idea, further analysis showed that the positive curvature was present in the control group (γ = 0.0038±0.0007 fF/(pC∧2), p<0.001, n = 13 cells) but not in the EGTA group (γ = 0.0003±0.0005 fF/(pC∧2), p = 0.49, n = 18 cells), which might be because of EGTA suppressing Ca^2+^ elevations and exocytosis mainly away from Ca^2+^ channels.

### Recovery of Ca^2+^ current inactivation is enough to reset the exocytotic response

To address the question whether pool depletion or Ca^2+^ channel inactivation is the cause of the declined exocytotic response from another perspective, we generated another set of data. In this experiment the protocol started with two depolarizations, prepulse plus pulse-length protocol, as in the previous experiment. The difference was that these two depolarizations were followed by a third 500-ms depolarization to 0 mV after a rest interval at −70 mV lasting either 200 ms (protocol I) or 10 seconds (protocol II). Our idea was based on the fact that the Ca^2+^ current recovers much faster [40] from inactivation than the RRP recovers from depletion, which takes almost a minute to refill [41]. If the first two pulses do not deplete the RRP, then current recovery would dictate the exocytotic response evoked by the third depolarization, and we should expect the Ca^2+^ current sensitivity between protocols I and II for the third depolarization to be equal, and Ca^2+^ current sensitivity similar to the pulse-length protocols investigated above. In contrast, if the current recovers in 10 seconds but the pool does not, then the Ca^2+^ current sensitivity should be lower for the third pulse in protocol II than for the pulse-length protocols. Moreover, the current would recover to some extent while the pool would not during a 200 ms rest period. Thus, the Ca^2+^ current sensitivity for the third pulse should be even lower in protocol I than in protocol II if the pool of granules is depleted.

**Figure 7:**
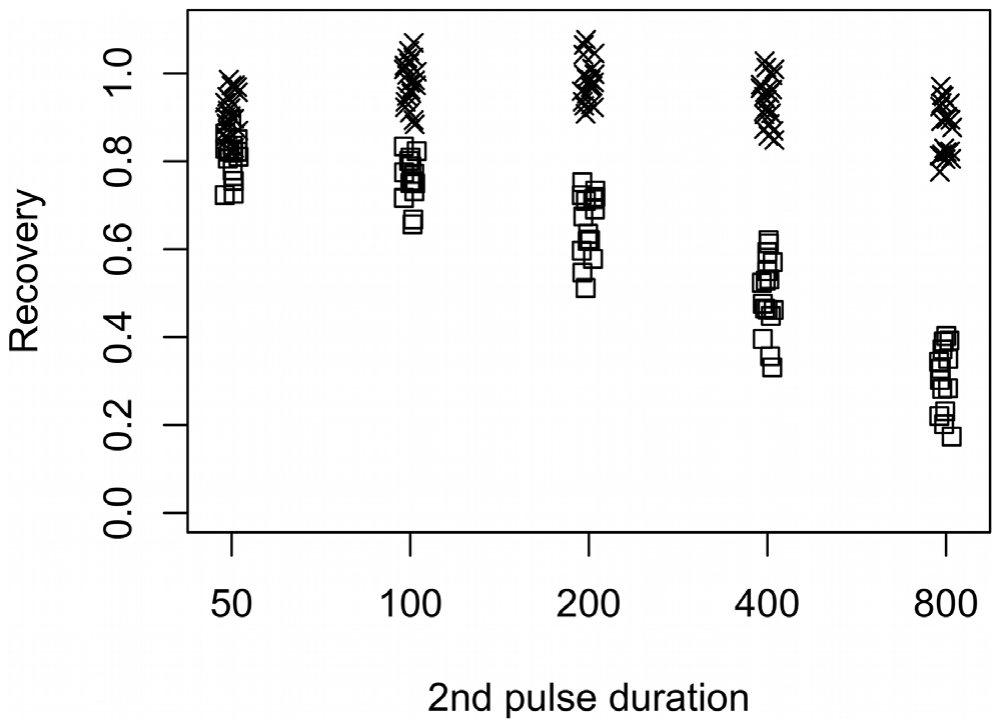
Current recovery *Q*_3,50 ms_/*Q*_1_ (see text) for the different durations of the second pulse and 200 ms (protocol I, squares) or 10 s (protocol II, crosses) resting period.

To investigate current recovery in our data, we calculated for each cell the amount of Ca^2+^ influx during the first 50 ms of the third pulse (*Q*_3,50 ms_), and related it to the Ca^2+^ influx during the 50 ms prepulse (*Q*_1_). Mean recovery was then defined as the average of the ratios *Q*_3,50 ms_/*Q*_1_. We found that following a 50 ms prepulse and a 50 or 100 ms second pulse, the Ca^2+^ current did not inactivate much, and hence recovered substantially (mean recovery >75%; Fig. 7) in just 200 ms. In contrast, following longer second pulses the current did not recover much during 200 ms resting period (protocol I, squares in Fig. 7), but recovered almost fully in 10 s (protocol II; crosses in Fig. 7). For example, following the 50 ms prepulse and 800 ms second pulse, mean current recovery was 0.31±0.02 in protocol I and 0.87±0.01 in protocol II. This is in agreement with investigations of Ca^2+^ current recovery in mouse beta-cells [40].

**Figure 8:**
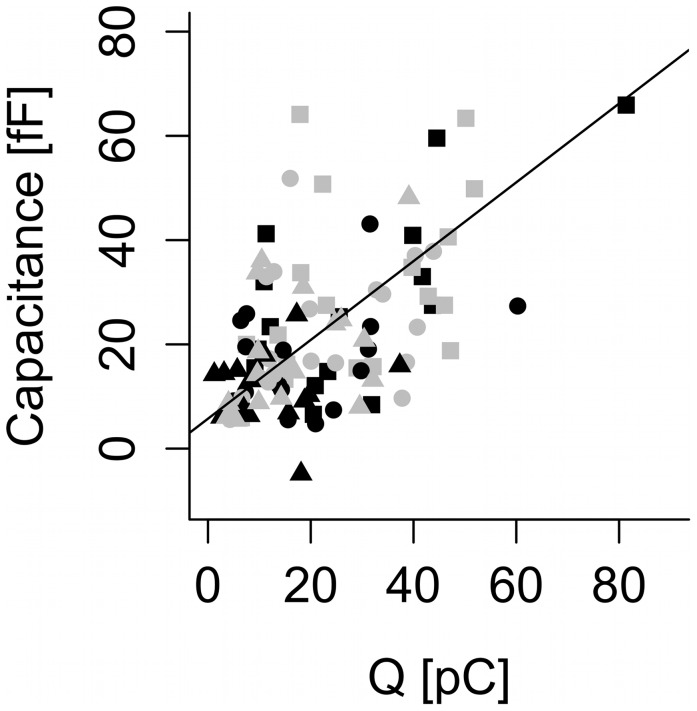
No difference in Ca^2+^ current sensitivity between protocol I and protocol II. Capacitance increases are plotted vs. Ca^2+^ influx *Q* for the third 500-ms depolarizations following a 50 ms prepulse, a second pulse of either 200 ms (squares), 400 ms (circles) or 800 ms (triangles), and a resting period of either 200 ms (protocol I, black) or 10 s (protocol II, gray). The line indicates the fixed-effects fit from the linear mixed-effects model, which fitted the entire data set (both protocols).

Since our argumentation is based on substantial Ca^2+^ current inactivation, we limited our analysis to third pulses following a 50-ms prepulse and a second depolarization lasting ≥200 ms. These longer pulses would also favor the unmasking of any pool depletion, if it should occur. Mixed-effects modeling with ΔC_m_ as a function of *Q* for third-pulse data with the second depolarization lasting ≥200 ms revealed no evidence of any difference between protocols I and II (p = 0.48 by likelihood ratio test, confirmed by the Akaike Information Criterion, between a model with intercept and slope depending on the protocol, and a simple model without protocol effect; Fig. 8). Moreover, the analysis gives results for the Ca^2+^ current sensitivity similar to the previous analyses of pulse-length data with or without prepulse (0.76±0.10 fF/pC). In summary, the exocytotic response recovers in parallel to Ca^2+^ currents, and the Ca^2+^ current sensitivity is unaffected by the two preceding pulses. These findings speak against pool depletion.

### A train of 500 ms depolarizations does not empty the RRP

Having established that short depolarizations deplete a small IRP of ∼10 fF, but do not exhaust subsequent exocytosis resulting from a larger pool, we analyzed if more intense stimulation, in the form of the widely used train protocol consisting of 500 ms pulses delivered at 1 Hz, could deplete the larger pool, likely the RRP.

Previous results [12] showed that in INS-1 832/13 cells the capacitance increases caused by the depolarizations do not diminish during the train. Here, we related the cumulative increase in membrane capacitance evoked by the depolarizations to the cumulative calcium influx with mixed-effects modeling. In case of pool depletion, we expect a concave, downward-curving, relation between cumulative capacitance and cumulative Ca^2+^ entry [22], as for the shorter pulses (note that the opposite, i.e. positive curvature, is to expect if the individual capacitance jumps is analyzed as a function of the calcium entry during each pulse [22]).

When analyzing all ten pulses of the train, we found no sign of pool depletion, and an intercept not different from zero (−3.1±15.1 fF, n = 7 cells, p = 0.84). The Ca^2+^ current sensitivity was estimated to be 1.81±0.48 fF/pC. However, when we analyzed the responses to the first three pulses only, it was clear that the data showed a non-zero intercept (29.1±8.6 fF, p = 0.005), because the first pulse released relatively more granules than the second and third pulses. This finding is in agreement with the IRP from the data analyzed in the previous sections. The Ca^2+^ current sensitivity was estimated to be 1.46±0.62 fF/pC for the first three pulses. Cell-to-cell variation was big with estimated standard deviations of the random effects amounting to 17.9 fF for the intercept, and 1.55 for the Ca^2+^ current sensitivity. In the full data set including all ten pulses the non-zero intercept was masqueraded by an upward curvature towards the end of the train, since a linear fit tends to lower the intercept and increase the slope in order to approximate the last data points. Our results are similar to the patterns observed in rat beta-cells stimulated by a train of 40 ms depolarizations delivered at 10 Hz [15].

## Discussion

In this work we have carefully studied the basic mechanisms underlying the coupling of Ca^2+^ entry to insulin release using an accepted model cell system, with the main finding that the RRP in INS-1 832/13 cells is not as easily depleted as previously thought, and that Ca^2+^ current inactivation can masquerade as pool depletion. INS-1 832/13 cells are a commonly used cell line in confirming human findings. We are most aware that cell lines are not primary cells and that there are large differences between murine and human beta-cells [9], [42]–[46]. However, the source of human and primary cells is limited, and accordingly to the 3Rs (http://www.nc3rs.org.uk) we should strive to work as much as possible in cell lines [47] and to use mathematical models [48]. Hence, besides providing valuable insight in the control of exocytosis in its own right, it is important to understand the basic mechanisms in the cell-line model to be aware of limitations when comparing with the human setting.

Using patch-clamp, capacitance measurements and mixed-effect modeling, we showed by three different protocols that pool depletion plays a negligible role in shaping the decline in the exocytotic response seen in INS-1 832/13 cells when ΔC_m_ is related to pulse duration (Fig. 1A). The observed ΔC_m_ profile is instead mostly determined by the kinetics of Ca^2+^ current inactivation (Fig. 1B and C). Modifying Ca^2+^-channel kinetics by mutating domain involved in inactivation, or pharmacologically e.g. with the Ca^2+^-channel agonist BayK [8] could provide further insight concerning the role of Ca^2+^ currents in shaping the exocytosis patterns. Interestingly, BayK did not modify the linear relation between ΔC_m_ and Q in mouse beta-cells [8].

In agreement with the lack of depletion in the pulse-length protocols, we found that intense stimulation by the train protocol did not deplete the RRP. These conclusions are in line with the fact that INS-1 832/13 cells show a slow, graded Ca^2+^ response to elevation in the glucose concentration [49] and the absence of biphasic insulin secretion [50], in contrast to the INS-1 mother cell line, which shows biphasic Ca^2+^ patterns and insulin release [51]. Thus, these previous findings [49]–[51] suggest that the difference between the two cell lines with respect to secretory dynamics can largely be explained by the different Ca^2+^ profiles with no need for pool depletion.

We found evidence of a tiny pool of granules of ∼7 fF, or ∼10 granules, that could be released my minimal amounts of entering Ca^2+^. It is therefore likely that this pool (IRP) is situated near Ca^2+^ channels, and is similar to IRP measured in murine cells, although ten times smaller in size. Our finding that EGTA does not interfere with release from this small pool supports this conclusion. Similarly, Yang and Gillis [52] found evidence of an IRP that was exhausted by 10 ms depolarizations in INS-1 cells.

In contrast, later exocytosis was strongly affected by EGTA, which lowered the Ca^2+^ current sensitivity of exocytosis by more than 50%. This suggests that in INS-1 832/13 cells most exocytosis occurs away from Ca^2+^ channels from a granule pool that is not depleted by depolarizations lasting even up to a second. Either this pool is very large or is very rapidly refilled. The exocytotic responses is of similar magnitude in INS-1 832/13 cells as in primary cells, but electron micrographs from insulinoma cells demonstrate that these cells have fewer granules and of smaller size [34], [53] as compared to primary cells [4], [17]. It is therefore most likely that INS-1 832/13 cells have machinery that requires a more rapid refilling from a large pool that cannot be depleted by the prolonged stimuli used here. Yang and Gillis [52] found that the RRP, as determined by flash-released Ca^2+^-induced exocytosis, is ∼100 fF in INS-1 cells. Accordingly, one half of this pool is released by a 640 ms depolarization (Fig. 1A), if we assume no refilling during the pulse. However, we favor the interpretation that refilling is rapid and occur during each depolarization.

**Figure 9:**
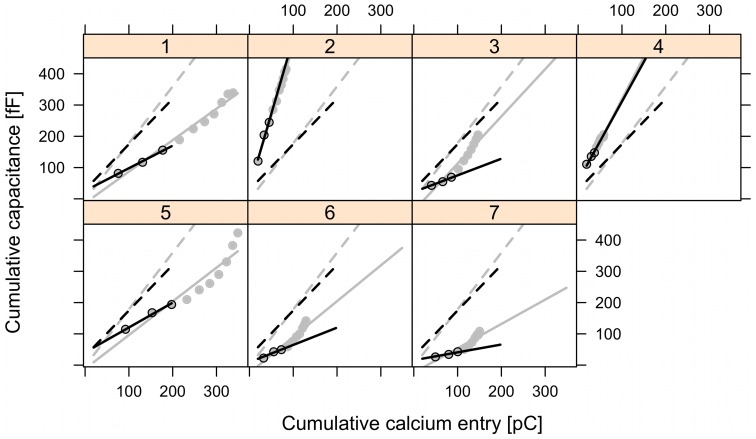
Mixed-effects analysis of the train protocol. Cumulative capacitance data is plotted against cumulative Ca^2+^ influx measured as charge, for individual cells with single-cell fits indicated by solid lines, while group fits (fixed-effects) are given by the dashed lines. Model fit to all cumulative data from all 10 pulses is given in gray, while the fit to the first three pulses is given in black.

Interestingly, and supporting our conclusion, repeated 500-ms pulses did not lead to the exhaustion of the increases in membrane capacitance in INS-1 832/13 cells (Fig. 9) [12]. However, we did find evidence of an exhaustible pool since our analysis of the first three pulses revealed a non-zero intercept ∼30 fF, which likely corresponds to the IRP found in the pulse-length and double-pulse protocols. It is not clear why the cells used for the train protocol have larger IRP, but the Ca^2+^ current sensitivity was also increased, suggesting that these cells were generally highly responsive. Note also that there was a large uncertainty in the estimate of the size of the IRP (standard error 8.6 fF) and a large cell-to-cell variation (the standard deviation of the random effect was 17.9 fF), indicating that the train protocol is not appropriate for determining the size of the IRP.

Late exocytosis was not reduced by the train of pulses, but rather tended to increase when Ca^2+^ entry was taken into account. This convex ΔC_m_ vs. *Q* relation could result for example from residual Ca^2+^ from the first pulses increasing the Ca^2+^ current sensitivity (less Ca^2+^ entry was needed to trigger exocytosis, since it was summarized on the residual Ca^2+^), or residual Ca^2+^ could increase the rate of docking and refilling, which are Ca^2+^ dependent processes. Moreover, in INS-1 cells a train of 500-ms depolarizations did not provoke pool depletion at temperatures similar to the ones used here, but at lower temperature (24°C) capacitance increases were markedly reduced after the first two pulses [54]. In the latter study, it was suggested that vigorous, random movement of granules are needed to sustain late exocytosis in INS-1 cells, in line with the findings and interpretations presented here.

Away from Ca^2+^ channels the Ca^2+^ concentration reaches a few μM [20], [46], [55]. Consequently, if most of exocytosis occurs away from Ca^2+^ channels, the fusing granules must be “highly Ca^2+^ sensitive” in order to be released. Yang and Gillis [52] described a highly Ca^2+^ sensitive pool (HCSP) in INS-1 cells, and it seems likely that the HCSP is also present in INS-1 832/13 cells and is responsible for most of the exocytosis seen in our experiments. Moreover, the data suggest that this pool in INS-1 832/13 cells not only have high Ca^2+^ sensitivity but is also difficult to deplete.

It has been suggested [56] that HCSP fusion corresponds to “newcomer granules”, i.e., granules that undergo exocytosis near-instantly when arriving at the plasma membrane [57]–[62]. Following this hypothesis, one would expect that most exocytosis in INS-1 832/13 cells is due to newcomers, as observed in INS-1 cells [62]. This would also provide a possible explanation of the lack of depletion seen in our experiments: if exocytosis occurs by newcomer fusion, then granules located away from the plasma membrane are releasable in addition to membrane-docked granules, which would allow for a larger total pool of releasable granules. This is also in agreement with ultrastructural data [53]. In this context it is worth noting that our experiments were done with cAMP in the patch pipette, and that cAMP has been shown to enhance both the HCSP [52] and newcomer fusion [59].

A linear relation between ΔC_m_ and *Q*, as observed in the INS-1 832/13 cells, is also seen in pulse-length data from mouse beta-cells [8], [20], indicative of the absence of pool depletion [22]. However, mouse islets have prominent biphasic insulin secretion patterns not only in response to high glucose, but also when stimulated by high extracellular concentrations of K^+^ [63]. This fact indicates a clear role for pool depletion in mouse beta-cells, which is supported by capacitance measurements showing a clear RRP in these cells [17], [41]. Similarly, human islets show biphasic insulin secretion when depolarized by sulfonylureas or K^+^ [64], [65], suggesting that pool depletion is important in human beta-cells. Correspondingly, capacitance measurements from human beta-cells in situ provide evidence of an IRP [21], and recordings from isolated human beta-cells reveal an RRP [19], [66].

In summary, we demonstrate that the release mechanism in INS-1 832/13 is different from primary cells, in particular that the RRP in INS-1 832/13 is not easily depleted. Our findings underline that the interplay between RRP depletion and phasic Ca^2+^ dynamics must be taken into account for a better elucidation of the biphasic nature of insulin secretion.

## Supporting Information

Data S1 **Pulse length protocol (5–640 ms) ΔC_m_ vs.** ***Q*** **data (** **Figs. 1** **and** **2** **).**
(DAT)

Data S2 **50 ms prepulse followed by pulse length protocol (50–800 ms) ΔC_m_ vs.** ***Q*** **data (** **Figs. 4** **and** **6** **).**
(DAT)

Data S3 **50 ms prepulse followed by pulse length protocol (50–800 ms) and a third 500 ms pulse after 200 ms or 10 s (** **Figs. 7** **and** **8** **).**
(DAT)

Data S4 **Train ΔC_m_ vs.** ***Q*** **data (** **Fig. 9** **).**
(DAT)
